# Supplementary material for: Exo70 Promotes the Invasion of Pancreatic Cancer Cells via the Regulation of Exosomes
Source: Cancers (Basel). 2024 Jan 12;16(2):336. doi: 10.3390/cancers16020336 (PMC10813805; doi:10.3390/cancers16020336)
Supplement: Supplementary file 1 [file cancers-16-00336-s001.zip › cancers-2768569-supplementary.pdf]

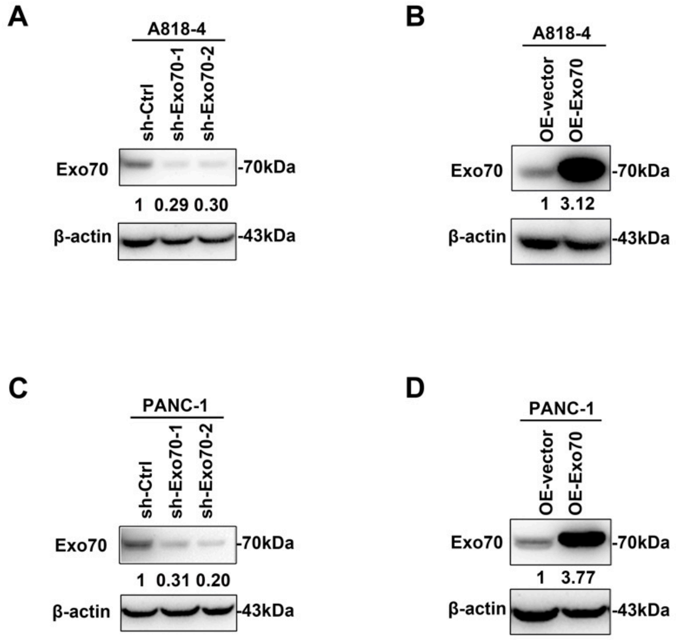

Supplemental Figure S1. PC cells (A818-4 and PANC-1) were transduced with lentivirus carrying shRNAs targeting both Exo70 or scramble control, or carrying exogenous Exo70 or the empty vector. (A, C) Knockdown of Exo70 was analyzed by immunoblot. (B, D) Overexpression of Exo70 was analyzed by immunoblot.

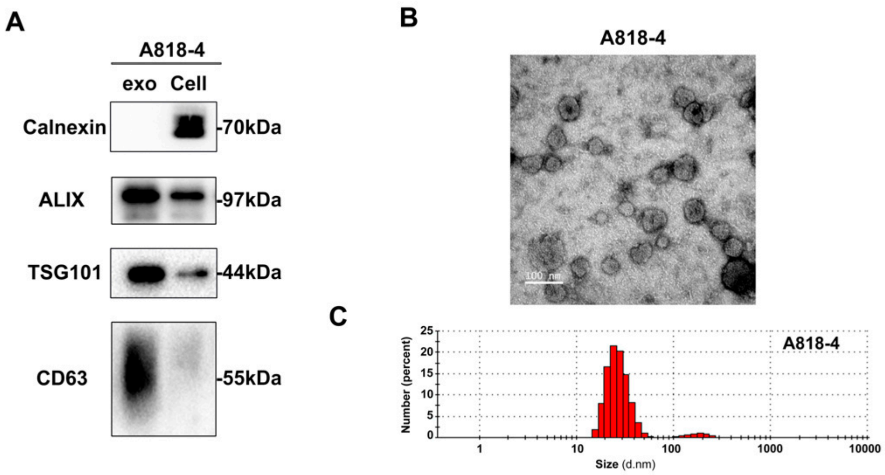

Supplemental Figure S2. Identification of exosomes and detection of exosome-related proteins in PC cells (A) (A) Western-blot detection of exosome marker protein markers in A818-4 cells. (B) Exosome morphology of A818-4 cells observed by transmission electron microscopy (C) Measurement of exosome particle size of A818-4 cells by NTA.

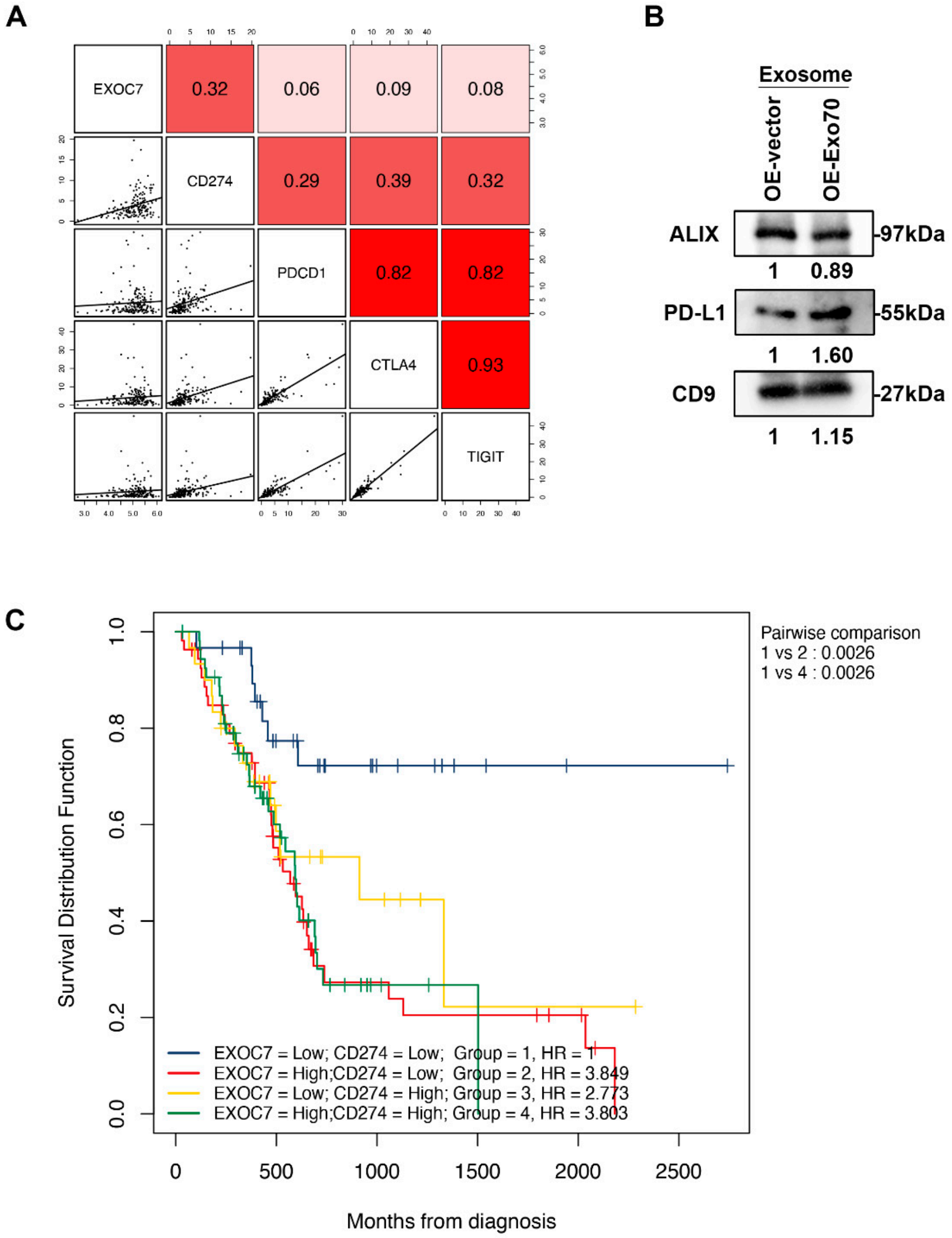

Supplemental Figure S3. Correlation between Exo70（EXOC7）and PD-L1. (A) TCGA database analysis of the correlation between Exo70 and immune checkpoints. (B) Western-blot detection of exosome marker protein markers and PD-L1 in OE-Exo70 exosomes. (C) Survival analysis of PC patients based on Exo70 and PD-L1 expression.

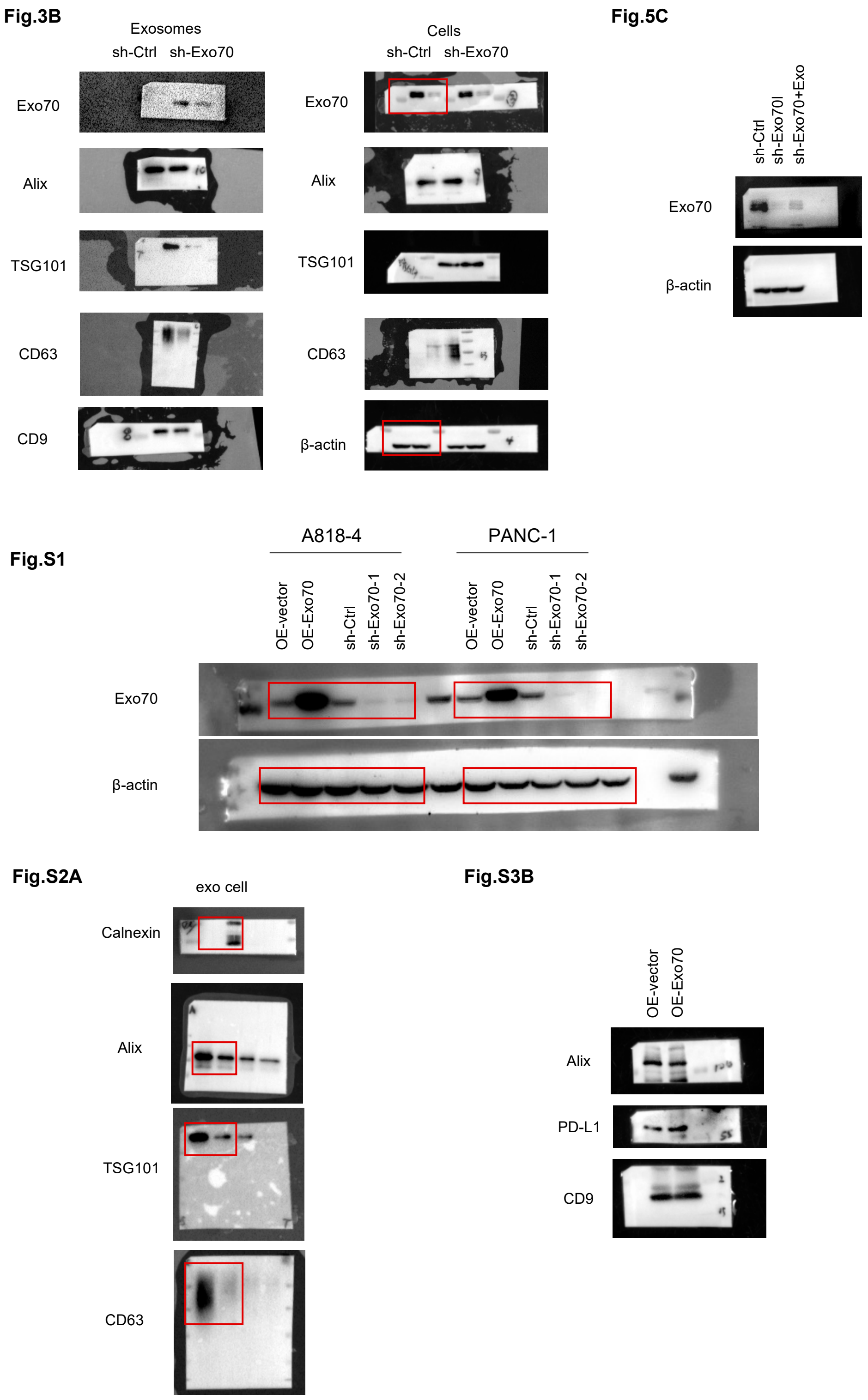

Supplemental Figure S4. Western blot analyses of all molecular in this paper (Fig.3B, Fig.5C, Fig.S1, Fig.S2A, Fig.S3B).
